# Effects of chalazia on corneal astigmatism: Large-sized chalazia in middle upper eyelids compress the cornea and induce the corneal astigmatism

**DOI:** 10.1186/s12886-017-0426-2

**Published:** 2017-03-31

**Authors:** Ki Won Jin, Young Joo Shin, Joon Young Hyon

**Affiliations:** 1 Department of Ophthalmology, Hallym University College of Medicine, Gangnam Sungshim Hospital, 948-1 Daerim1-dong, Youngdeungpo-gu, Seoul, 150-950 South Korea; 2 Department of Ophthalmology, Seoul National University College of Medicine, Seoul, South Korea; 3 Department of Ophthalmology, Seoul National University Bundang Hospital, Seongnam, Gyeonggi-do South Korea

**Keywords:** Chalazia, Astigmatism, Wavefront, Corneal topography

## Abstract

**Background:**

A chalazion is a common eyelid disease that causes eye morbidity due to inflammation and cosmetic disfigurement. Corneal topographic changes are important factors in corneal refractive surgery, intraocular lens power calculations for cataract surgery, and visual acuity assessments. However, the effects of chalazia on corneal astigmatism have not been thoroughly investigated. The changes in corneal astigmatism according to chalazion size and location is necessary for better outcome of ocular surgery. The aim of this study is to evaluate changes in corneal astigmatism according to chalazion size and location.

**Methods:**

In this cross-sectional study, a total of 44 eyes from 33 patients were included in the chalazion group and 70 eyes from 46 patients comprised the control group. Chalazia were classified according to location and size. An autokeratorefractometer (KR8100, Topcon; Japan) and a Galilei™ dual-Scheimpflug analyzer (Ziemer Group; Port, Switzerland) were utilized to evaluate corneal changes.

**Result:**

Oblique astigmatism was greater in the chalazion group compared with the control group (*p* < 0.05). Astigmatism by simulated keratometry (simK), steep K by simK, total root mean square, second order aberration, oblique astigmatism, and vertical astigmatism were significantly greater in the upper eyelid group (*p* < 0.05). Astigmatism by simK, second order aberration, oblique astigmatism, and vertical astigmatism were significantly greater in the large-sized chalazion group (*p* < 0.05). Corneal wavefront aberration was the greatest in the upper eyelid chalazion group, whole area group, and large-sized chalazion group (*p* < 0.05).

**Conclusions:**

Large-sized chalazia in the whole upper eyelid should be treated in the early phase because they induced the greatest change in corneal topography. Chalazion should be treated before corneal topography is performed preoperatively and before the diagnosis of corneal diseases.

**Electronic supplementary material:**

The online version of this article (doi:10.1186/s12886-017-0426-2) contains supplementary material, which is available to authorized users.

## Background

A chalazion is a meibomian gland lipogranuloma which accompanies swelling on the eyelid and eyelid tenderness [1]. It is a common eyelid disease that causes eye morbidity due to inflammation and cosmetic disfigurement [2]. A variety of factors are believed to be associated with the development of chalazia including meibomian gland dysfunction, chronic blepharitis, seborrheic dermatitis, gastritis, and smoking [1]. Chalazia treatment includes medical treatments, such as warm compression and topical antibiotic eye drops or ointment, and surgical incision and curettage, with or without triamcinolone intralesional injection [3].

Corneal topographic changes are important factors in corneal refractive surgery, intraocular lens power calculations for cataract surgery, and visual acuity assessments [4–6]. In addition, amblyopia may develop in children with corneal astigmatism [7]. It has been reported that the pressure of an upper lid chalazion induces hyperopia and astigmatism.7 Chalazia can increase higher-order aberrations (HOAs), as measured by the Hartmann–Shack aberrometer; these can affect the preoperative evaluation and refractive surgery outcomes, especially wavefront-guided approaches [8]. In addition, decreased vision due to a chalazion of the upper eyelid has been documented in a patient following laser-assisted in situ keratomileusis (LASIK) [9]. Furthermore, corneal aberration has been reported to contribute to the visual function [10, 11]. The changes in corneal astigmatism according to chalazion size and location is necessary for better outcome of ocular surgery.

However, the effects of chalazia on corneal astigmatism have not been thoroughly investigated. In this study, we investigated changes in corneal astigmatism according to chalazion size and location.

## Methods

This study adhered to the tenets of the Declaration of Helsinki and was approved by the Institutional Review Board of Hallym University Medical Center. Medical charts of a total of 114 eyes from 64 patients were reviewed retrospectively in this study between July 2013 and April 2015 at the Hallym University Gangnam Sacred Heart Hospital, Seoul, South Korea. Forty four eyes from 33 patients exhibiting an eyelid chalazion were assigned to the chalazion group. The control group comprised 22 contralateral normal eyes of chalazion patients and 48 eyes from 24 patients without a chalazion, randomly selected and matched for age and sex. Patient medical history including diabetes mellitus and hypertension was obtained and a physical examination of eye and eyelid was performed prior to study procedures. Patients in the control group did not have a history of ophthalmic surgery including eyelid surgery and were not using topical or systemic medications on examination.

Chalazia were classified according to their site (upper, lower, or both eyelid groups) and location (nasal, middle, temporal, or whole area of eyelid). They also were classified into groups according to their size; small (≤1/5 of eyelid), medium (2/5–3/5), or large (>4/5).

An autokeratorefractometer (ARK; KR8100, Topcon; Japan) was utilized to measure keratometric values (K) including mean K, flat and steep K, astigmatism, and axis. Central corneal thickness (CCT), corneal topographic data, and wavefront aberration data were obtained using a Galilei™ dual-Scheimpflug analyzer (Ziemer Group; Port, Switzerland). Simulated K (simK) were obtained from the central 3-mm zone of the corneas including flat and steep K, mean K, astigmatism (difference between steep and flat Ks), and the axis of the steep meridian.

Corneal wavefront aberrations were analyzed, including total root mean square (RMS, in microns) of the total high order aberration, second order aberration, oblique astigmatism (Z^−2^_2_), defocus (Z^0^_2_), vertical astigmatism (Z^2^_2_), third order aberration, vertical trefoil (Z^−3^_3_), vertical coma (Z^−1^_3_), horizontal coma (Z^1^_3_), oblique trefoil (Z^3^_3_), fourth order aberration, oblique quadrefoil (Z^−4^_4_), secondary oblique astigmatism (Z^−2^_4_), primary spherical aberration (Z^0^_4_), vertical secondary astigmatism (Z^2^_4_), and vertical quadrefoil (Z^4^_4_).

### Statistical analysis

All statistical analyses were performed using SPSS v.18.0 (IBM Corp., NY, USA). An independent *t*-test was used to compare the outcomes between the chalazion and control groups. Analysis of variance, followed by Tukey post hot test, was performed to determine differences between subgroups.

## Results

A total 114 eyes from 64 patients were included in this study: 44 eyes in the chalazion group and 70 eyes in the control group (Table 1). Mean patient age was 40.0 ± 13.9 years in the chalazion group and 43.4 ± 14.0 years in the control group. The chalazion group was divided into the following subgroups: 1) according to site of the chalazion, the upper eyelid (*n* = 22), lower eyelid (*n* = 16), and both eyelids (*n* = 6), 2) according to the location of the chalazion, the nasal eyelid (*n* = 10), middle eyelid (*n* = 25), temporal eyelid (*n* = 4), and whole eyelid (*n* = 3), and 3) according to the size of the chalazion, small (*n* = 14), medium (*n* = 17), and large (*n* = 11) (Additional file 1).

**Table 1:** Demographic data of subjects

	N
Control	70
Chalazion group	44
Site	
Upper eyelid	22
Lower eyelid	16
Both eyelid	6
Location	
Nasal	10
Middle	25
Temporal	4
Whole	3
Size	
Small	14
Medium	17
Large	11

**Fig. 1:**
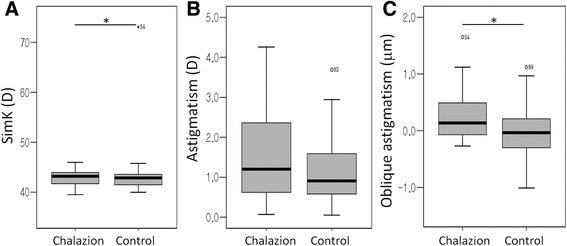
Corneal topographic data for the chalazion and control groups. Simulated K (simK; (**a**) and astigmatism by simK (**b**) is similar between the two groups. Oblique astigmatism (Z^−2^_2_; **c**) is greater in the chalazion group compared with the control group (*p* = 0.013; independent *t*-test)

Corneal topographic data for the chalazion and control groups are presented in Fig. 1 and Table 2. There was no difference in CCT different between the two groups. Astigmatism measured by ARK was not significantly different between the chalazion and control groups (*p* = 0.074; independent *t*-test). Oblique astigmatism (Z^−2^_2_) was greater in the chalazion group compared with the control group (*p* = 0.013; independent *t*-test). Other topographic data were similar between the chalazion and control groups.

**Table 2:** Corneal topographic data between chalazion and control group

	Total	Chalazion group	Control group	*p*-value
N (eyes)	114	44	70	
Gender (M:F)	52:62	19:25	33:37	
Age (year)	41.59 ± 14.08	39.57 ± 13.83	42.86 ± 14.18	0.226
CCT (μm)	547.25 ± 39.90	546.91 ± 43.64	547.46 ± 37.69	0.943
Average keratometry by ARK (D)	42.96 ± 1.86	42.84 ± 2.08	43.03 ± 1.72	0.603
Astigmatism by ARK (D)	-0.85 ± 0.99	−0.94 ± 1.44	−0.79 ± 0.58	0.546
Axis by ARK (°)	104.23 ± 63.36	108.63 ± 60.74	101.48 ± 65.26	0.579
SimK (D)	42.76 ± 3.49	42.43 ± 2.28	42.96 ± 4.08	0.434
Astigmatism by simK (D)	1.31 ± 0.96	1.53 ± 1.16	1.17 ± 0.78	0.074
Axis by simK (°)	84.74 ± 35.24	85.16 ± 28.23	84.47 ± 39.20	0.914
Mean K of posterior surface (D)	−6.28 ± 0.27	−6.25 ± 0.24	−6.29 ± 0.28	0.514
Astigmatism of posterior surface (D)	−0.44 ± 0.29	−0.46 ± 0.26	−0.43 ± 0.32	0.691
Total RMS (μm)	1.81 ± 0.80	1.97 ± 1.05	1.71 ± 0.59	0.127
2nd order aberration (μm)	1.55 ± 0.70	1.68 ± 0.87	1.48 ± 0.55	0.184
Oblique astigmatism (Z^−2^_2_; μm)	0.04 ± 0.49	0.18 ± 0.52	−0.05 ± 0.45	0.013*
Defocus (Z^0^_2_; μm)	−0.85 ± 0.50	−0.83 ± 0.53	−0.87 ± 0.49	0.693
Vertical astigmatism (Z^2^_2_; μm)	−0.74 ± 1.06	−0.98 ± 1.16	−0.59 ± 0.98	0.057
3rd order aberration (μm)	0.67 ± 0.42	0.71 ± 0.53	0.64 ± 0.34	0.398
Vertical trefoil (Z^−3^_3_; μm)	−0.18 ± 0.40	−0.24 ± 0.44	−0.14 ± 0.37	0.216
Vertical Coma (Z^−1^_3_; μm)	0.34 ± 3.01	0.13 ± 0.37	0.48 ± 3.84	0.555
Horizontal coma (Z^1^_3_; μm)	−0.04 ± 0.31	−0.04 ± 0.29	−0.04 ± 0.33	0.961
Oblique trefoil (Z^3^_3_; μm)	−0.02 ± 0.44	−0.06 ± 0.55	−0.01 ± 0.35	0.378
4th order aberration (μm)	0.40 ± 0.30	0.40 ± 0.30	0.40 ± 0.30	0.921
Oblique quadrefoil (Z^−4^_4_; μm)	0.01 ± 0.09	0.02 ± 0.10	0.00 ± 0.07	0.293
Oblique secondary astigmatism (Z^−2^_4_; μm)	0.01 ± 0.13	−0.01 ± 0.14	0.01 ± 0.11	0.333
Primary spherical (Z^0^_4_; μm)	0.17 ± 0.30	0.17 ± 0.32	0.16 ± 0.30	0.922
Vetical secondary astigmatism (Z^2^_4_; μm)	0.07 ± 0.18	0.05 ± 0.19	0.08 ± 0.18	0.447
Vertical quadrefoil (Z^4^_4_; μm)	−0.11 ± 0.23	−0.11 ± 0.22	−0.12 ± 0.24	0.888

*SimK* simulated keratometry, *ARK* autorefractokeratometry, *RMS* root mean square, *D* diopter; *Statistically significant by independent *t*-test

**Fig. 2:**
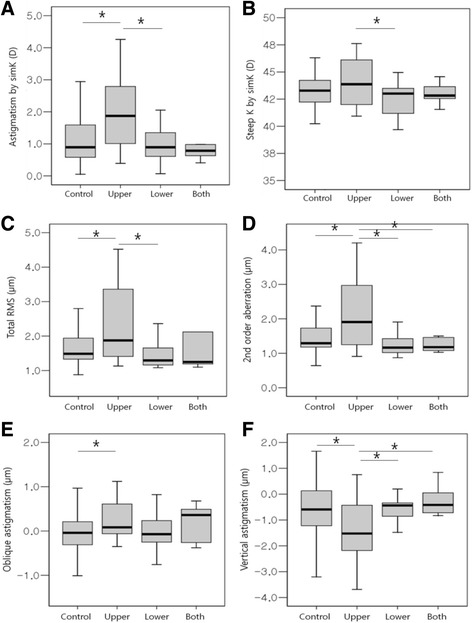
Corneal topographic data according to the site of chalazion. Chalazia are classified into control, upper, lower, or both eyelid group. Astigmatism by simulated keratometry (simK; (**a**), steep keratometry (K) by simK (**b**), total root mean square (RMS; **c**), second order aberration (**d**), oblique astigmatism (Z^−2^_2_; **e**), and vertical astigmatism (Z^2^_2_; **f**) are significantly different between the subgroups (*p* = 0.001, 0.022, 0.002, < 0.001, 0.009, and 0.001, respectively; one-way analysis of variance)

The CCT was not significantly different between the chalazion site subgroups (Fig. 2, Table 3). However, astigmatism by simK, steep K by simK, total RMS, second order aberration, Z^−2^_2_, and Z^2^_2_ were significantly different between these subgroups (*p* = 0.001, 0.022, 0.002, <0.001, 0.009, and 0.001, respectively; ANOVA). Astigmatism by simK was greater in the upper eyelid group compared with the control and lower eyelid groups (*p* = 0.001 and 0.004, respectively; Tukey post hoc test). Steep K by simK significantly differed between upper and lower lids (*p* = 0.011; Tukey post hoc test). Total RMS was greater in the upper eyelid group compared with the control and lower eyelid groups (*p* = 0.004 and 0.003, respectively; Tukey post hoc test). Second order aberration was greater in the upper eyelid group compared with the control, lower eyelid, and whole eyelid groups (*p* = 0.001, <0.001, and 0.019, respectively; Tukey post hoc test). The Z^−2^_2_ was greater in the upper eyelid group compared with the control (*p* = 0.06, Tukey post hoc test). The Z^2^_2_ was greater in the upper eyelid group compared with the control and lower eyelid group, and lower in the upper eyelid group compared with whole eyelid group (*p* = 0.002, 0.008 and, 0.028, respectively; Tukey post hoc test).

**Table 3:** Corneal topographic data according to site of chalazion

	Control	Upper eyelid	Lower eyelid	Both eyelids	*p*-value
n	70	22	16	6	
Gender (M:F)	33:37	10:12	3:13	6:0	
Age (year)	42.86 ± 14.18	41.27 ± 12.41	38.63 ± 16.74	35.83 ± 11.16	0.519
CCT (μm)	547.46 ± 37.69	5583.27 ± 42.50	528.25 ± 45.57	555.00 ± 28.33	0.136
Average keratometry by ARK (D)	43.03 ± 1.72	43.38 ± 1.69	42.18 ± 2.58	42.82 ± 1.44	0.269
Astigmatism by ARK (D)	-0.79 ± 0.58	−1.12 ± 1.93	−0.88 ± 0.85	−0.50 ± 0.45	0.490
Axis by ARK (°)	101.48 ± 65.26	118.16 ± 67.99	102.50 ± 53.94	92.00 ± 57.73	0.750
SimK (D)	42.96 ± 4.08	43.11 ± 1.69	41.51 ± 2.99	42.42 ± 1.21	0.470
Astigmatism by simK (D)	1.17 ± 0.78	2.01 ± 1.27	0.98 ± 0.59	1.23 ± 1.26	0.001*
Axis by simK (°)	84.47 ± 39.20	83.05 ± 25.31	88.31 ± 27.04	84.50 ± 43.78	0.470
Mean K of posterior surface (D)	−6.29 ± 0.28	−6.29 ± 0.29	−6.24 ± 0.15	−6.16 ± 0.25	0.653
Astigmatism of posterior surface (D)	−0.43 ± 0.32	−0.53 ± 0.32	−0.36 ± 0.13	−0.42 ± 0.16	0.336
Total RMS (μm)	1.71 ± 0.59	2.35 ± 1.13	1.46 ± 0.39	1.96 ± 1.47	0.002*
2nd order aberration (μm)	1.48 ± 0.55	2.11 ± 1.04	1.23 ± 0.31	1.24 ± 0.20	<0.001*
Oblique astigmatism (Z^−2^_2_; μm)	−0.05 ± 0.45	0.33 ± 0.57	−0.03 ± 0.40	0.21 ± 0.43	0.009*
Defocus (Z^0^_2_; μm)	−0.87 ± 0.49	−0.79 ± 0.72	−0.82 ± 0.25	−0.99 ± 0.10	0.820
Vertical astigmatism (Z^2^_2_; μm)	−0.59 ± 0.98	−1.55 ± 1.28	−0.48 ± 0.69	−0.25 ± 0.62	0.001*
3rd order aberration (μm)	0.64 ± 0.34	0.85 ± 0.68	0.55 ± 0.17	0.62 ± 0.43	0.129
Vertical trefoil (Z^−3^_3_; μm)	−0.14 ± 0.37	−0.28 ± 0.54	−0.16 ± 0.21	−0.31 ± 0.50	0.470
Vertical Coma (Z^−1^_3_; μm)	0.48 ± 3.84	0.23 ± 0.41	−0.02 ± 0.31	0.18 ± 0.30	0.939
Horizontal coma (Z^1^_3_; μm)	−0.04 ± 0.33	−0.025 ± 0.29	−0.13 ± 0.31	0.13 ± 0.15	0.398
Oblique trefoil (Z^3^_3_; μm)	−0.01 ± 0.35	−0.16 ± 0.72	−0.01 ± 0.28	0.14 ± 0.30	0.332
4th order aberration (μm)	0.40 ± 0.30	0.44 ± 0.21	0.39 ± 0.40	0.30 ± 0.27	0.802
Oblique quadrefoil (Z^−4^_4_; μm)	0.00 ± 0.07	0.03 ± 0.14	0.02 ± 0.05	−0.02 ± 0.03	0.422
Oblique secondary astigmatism (Z^−2^_4_; μm)	0.01 ± 0.11	−0.02 ± 0.16	−0.00 ± 0.11	0.03 ± 0.15	0.618
Primary spherical (Z^0^_4_; μm)	0.16 ± 0.30	0.10 ± 0.23	0.29 ± 0.43	0.11 ± 0.09	0.243
Vetical secondary astigmatism (Z^2^_4_; μm)	0.08 ± 0.18	0.10 ± 0.21	−0.01 ± 0.16	0.02 ± 0.06	0.230
Vertical quadrefoil (Z^4^_4_; μm)	−0.12 ± 0.24	−0.13 ± 0.25	−0.07 ± 0.11	−0.15 ± 0.33	0.876

*SimK* simulated keratometry, *ARK* autorefractokeratometry, *RMS* root mean square, *D* diopter; Results were presented as mean ± standard deviation

*Statistically significant by ANOVA

**Fig. 3:**
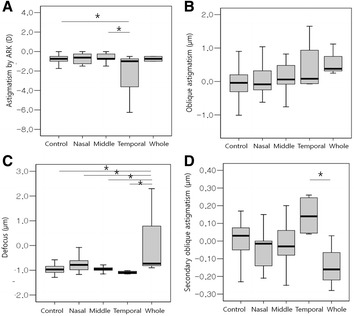
Corneal topographic changes according to the chalazion location. Chalazia are classified into control, nasal, middle, temporal, or whole area group. Astigmatism by auto-refractokeratometer (**a**), oblique astigmatism (Z^−2^_2_; **b**), defocus (Z^0^_2_; **c**), and secondary oblique astigmatism (Z^−2^_4_; **d**) are significantly different between groups (*p* = 0.046, 0.033, 0.003, and 0.015, respectively, one-way analysis of variance)

Corneal topographic changes according to chalazion location are presented in Fig. 3 and Table 4. The CCT was also not significantly different between chalazion location subgroups. Astigmatism by ARK, Z^−2^_2_, Z^0^_2_, and Z^−2^_4_ were significantly different between groups (*p* = 0.046, 0.033, 0.003, and 0.015, respectively; ANOVA). Astigmatism by ARK was significantly different between the control and temporal area groups or between middle and temporal area group (*p* = 0.019 and 0.025; Tukey post hoc test). The Z^0^_2_ was greater in the whole area group compared with the control, nasal, middle, and temporal area groups (*p* = 0.002, 0.021, 0.001, and 0.004, respectively; Tukey post hoc test). There was a significant difference in Z^−2^_4_ between temporal and whole area groups (*p* = 0.018; Tukey post hoc test).

**Table 4:** Corneal topographic changes according to chalazion location

	Control	Nasal	Middle	Temporal	Whole	*p*-value
n	70	10	25	4	3	
Gender (M:F)	33:37	5:5	9:16	2:2	2:1	
Age (year)	42.86 ± 14.18	42.20 ± 16.29	38.12 ± 13.66	43.75 ± 14.48	45.67 ± 4.51	0.679
CCT (μm)	547.46 ± 37.69	550.50 ± 19.60	542.16 ± 50.65	542.25 ± 32.40	552.33 ± 14.05	0.952
Average keratometry by ARK (D)	43.03 ± 1.72	42.44 ± 1.71	42.83 ± 2.35	44.79 ± 1.51	42.51 ± 0.88	0.411
Astigmatism by ARK (D)	-0.79 ± 0.58	−0.93 ± 1.01	−0.79 ± 58.50	−2.58 ± 3.19	−0.75 ± 0.35	0.046*
Axis by ARK (°)	101.48 ± 65.26	108.89 ± 61.53	115.00 ± 58.50	126.67 ± 70.77	105.00 ± 49.50	0.851
SimK (D)	42.96 ± 4.08	41.96 ± 1.91	42.33 ± 2.59	43.47 ± 1.62	43.86 ± 2.15	0.823
Astigmatism by simK (D)	1.17 ± 0.78	1.16 ± 1.14	1.54 ± 1.13	1.61 ± 1.79	2.30 ± 0.56	0.143
Axis by simK (°)	84.47 ± 39.20	80.00 ± 33.72	82.92 ± 27.77	103.00 ± 29.06	92.00 ± 22.54	0.843
Mean K of posterior surface (D)	−6.29 ± 0.28	−6.19 ± 0.20	−6.24 ± 0.24	−6.40 ± 0.34	−6.42 ± 0.37	0.543
Astigmatism of posterior surface (D)	−0.43 ± 0.32	−0.43 ± 0.13	−0.45 ± 0.32	−0.46 ± 0.19	−0.58 ± 0.13	0.942
Total RMS (μm)	1.71 ± 0.59	1.64 ± 0.76	2.03 ± 1.14	2.09 ± 1.48	2.37 ± 1.05	0.243
2nd order aberration (μm)	1.48 ± 0.55	1.35 ± 0.78	1.69 ± 0.82	1.90 ± 1.53	2.14 ± 0.93	0.219
Oblique astigmatism (Z^−2^_2_; μm)	−0.05 ± 0.45	0.06 ± 0.48	0.17 ± 0.49	0.44 ± 0.82	0.58 ± 0.47	0.033*
Defocus (Z^0^_2_; μm)	−0.87 ± 0.49	−0.75 ± 0.34	−0.94 ± 0.15	−1.09 ± 0.06	0.22 ± 1.80	0.003*
Vertical astigmatism (Z^2^_2_; μm)	−0.59 ± 0.98	−0.56 ± 1.11	−1.00 ± 1.19	−1.18 ± 1.68	−1.51 ± 0.54	0.269
3rd order aberration (μm)	0.64 ± 0.34	0.65 ± 0.36	0.75 ± 0.66	0.67 ± 0.19	0.71 ± 0.30	0.877
Vertical trefoil (Z^−3^_3_; μm)	−0.14 ± 0.37	−0.27 ± 0.40	−0.31 ± 0.47	−0.01 ± 0.24	0.11 ± 0.51	0.212
Vertical Coma (Z^−1^_3_; μm)	0.48 ± 3.84	0.18 ± 0.24	0.16 ± 0.41	−0.12 ± 0.39	0.10 ± 0.46	0.986
Horizontal coma (Z^1^_3_; μm)	−0.04 ± 0.33	−0.07 ± 0.26	−0.05 ± 0.28	0.15 ± 0.47	0.00 ± 0.24	0.820
Oblique trefoil (Z^3^_3_; μm)	−0.01 ± 0.35	−0.12 ± 0.42	−0.13 ± 0.64	0.29 ± 0.19	0.10 ± 0.24	0.349
4th order aberration (μm)	0.40 ± 0.30	0.43 ± 0.20	0.38 ± 0.34	0.36 ± 0.07	0.56 ± 0.50	0.885
Oblique quadrefoil (Z^−4^_4_; μm)	0.00 ± 0.07	0.03 ± 0.14	0.03 ± 0.10	0.01 ± 0.01	−0.05 ± 0.06	0.380
Oblique secondary astigmatism (Z^−2^_4_; μm)	0.01 ± 0.11	−0.03 ± 0.12	−0.02 ± 0.12	0.15 ± 0.12	−0.14 ± 0.16	0.015*
Primary spherical (Z^0^_4_; μm)	0.16 ± 0.30	−0.03 ± 0.12	−0.02 ± 0.12	0.20 ± 0.14	−0.11 ± 0.50	0.590
Vetical secondary astigmatism (Z^2^_4_; μm)	0.08 ± 0.18	0.17 ± 0.26	0.20 ± 0.34	−0.03 ± 0.15	0.12 ± 0.41	0.754
Vertical quadrefoil (Z^4^_4_; μm)	−0.12 ± 0.24	0.03 ± 0.25	0.05 ± 0.12	0.01 ± 0.20	−0.18 ± 0.39	0.710

*SimK* simulated keratometry, *ARK* autorefractokeratometry, *RMS* root mean square, *D* diopter; Results were presented as mean ± standard deviation.; *Statistically significant by ANOVA

**Fig. 4:**
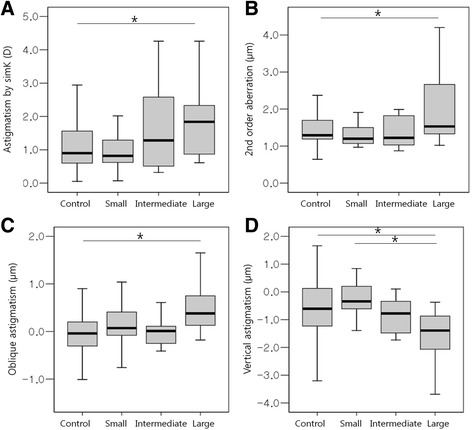
Corneal topographic changes according to chalazia size. Chalazia are classified into control, small-, medium- or large-sized groups. Astigmatism by simulated keratometry (simK; **a**), second order aberration (**b**), oblique astigmatism (Z^-2^_2_; **c**), and vertical astigmatism (Z^2^_2_; **d**) are significantly greater in the large-sized chalazion group (*p* = 0.037, 0.036, 0.006, and 0.002, respectively; one-way analysis of variance)

Corneal topographic changes according to chalazion size are presented in Fig. 4 and Table 5. The CCT was not significantly different between chalazion size subgroups. Astigmatism by simK, second order aberration, Z^−2^_2_, and Z^2^_2_ were greater in the large-sized chalazion group (*p* = 0.037, 0.036, 0.006, and 0.002, respectively; ANOVA). Astigmatism by simK and second order aberration was greater in the large-sized chalazion group compared with the control (*p* = 0.049 for both; Tukey post hoc test). There was a significantly greater Z^−2^_2_ in the large-sized chalazion group compared with the control (*p* = 0.003; Tukey post hoc test). Z^2^_2_ was greater in the large-sized chalazion group compared with the control and small-sized chalazion groups (*p* = 0.015 and 0.004, respectively; Tukey post hoc test).

**Table 5:** Corneal topographic changes according to chalazion size

	Control	Small	Medium	Large	*p*-value
n	70	14	17	11	
Gender (M:F)	33:37	5:9	6:11	7:4	
Age (year)	42.86 ± 14.18	43.64 ± 19.08	38.47 ± 10.72	38.36 ± 10.00	0.543
CCT (μm)	547.46 ± 37.69	539.29 ± 27.84	555.29 ± 34.43	535.91 ± 60.86	0.526
Average keratometry by ARK (D)	43.03 ± 1.72	43.66 ± 1.01	42.71 ± 2.11	42.09 ± 2.96	0.224
Astigmatism by ARK (D)	-0.79 ± 0.58	−0.85 ± 0.88	−0.89 ± 1.67	−1.20 ± 1.82	0.688
Axis by ARK (°)	101.48 ± 65.26	108.33 ± 50.24	113.75 ± 66.37	121.00 ± 55.42	0.714
SimK (D)	42.96 ± 4.08	43.07 ± 1.11	42.23 ± 2.30	42.05 ± 3.41	0.767
Astigmatism by simK (D)	1.17 ± 0.78	1.05 ± 0.67	1.69 ± 1.43	1.82 ± 1.13	0.037*
Axis by simK (°)	84.47 ± 39.20	89.93 ± 39.47	80.76 ± 26.52	84.45 ± 13.91	0.917
Mean K of posterior surface (D)	−6.29 ± 0.28	−6.23 ± 0.19	−6.25 ± 0.27	−6.30 ± 0.29	0.858
Astigmatism of posterior surface (D)	−0.43 ± 0.32	−0.34 ± 0.12	−0.54 ± 0.36	−0.48 ± 0.14	0.322
Total RMS (μm)	1.71 ± 0.59	1.77 ± 0.96	1.90 ± 1.05	2.33 ± 1.24	0.113
2nd order aberration (μm)	1.48 ± 0.55	1.34 ± 0.37	1.68 ± 1.04	2.06 ± 1.00	0.036*
Oblique astigmatism (Z^−2^_2_; μm)	−0.05 ± 0.45	0.09 ± 0.48	0.09 ± 0.50	0.49 ± 0.55	0.006*
Defocus (Z^0^_2_; μm)	−0.87 ± 0.49	−0.89 ± 0.31	−0.94 ± 0.15	−0.56 ± 0.96	0.222
Vertical astigmatism (Z^2^_2_; μm)	−0.59 ± 0.98	−0.20 ± 0.87	−1.15 ± 1.21	−1.60 ± 1.01	0.002*
3rd order aberration (μm)	0.64 ± 0.34	0.63 ± 0.30	0.64 ± 0.44	0.94 ± 0.84	0.169
Vertical trefoil (Z^−3^_3_; μm)	−0.14 ± 0.37	−0.24 ± 0.34	−0.23 ± 0.43	−0.26 ± 0.60	0.691
Vertical Coma (Z^−1^_3_; μm)	0.48 ± 3.84	0.01 ± 0.37	0.18 ± 0.42	0.22 ± 0.30	0.947
Horizontal coma (Z^1^_3_; μm)	−0.04 ± 0.33	−0.11 ± 0.32	0.03 ± 0.24	−0.01 ± 0.33	0.632
Oblique trefoil (Z^3^_3_; μm)	−0.01 ± 0.35	−0.04 ± 0.28	−0.02 ± 0.35	−0.19 ± 0.98	0.619
4th order aberration (μm)	0.40 ± 0.30	0.29 ± 0.18	0.38 ± 0.21	0.58 ± 0.45	0.094
Oblique quadrefoil (Z^−4^_4_; μm)	0.00 ± 0.07	0.04 ± 0.12	−0.00 ± 0.05	0.05 ± 0.14	0.296
Oblique secondary astigmatism (Z^−2^_4_; μm)	0.01 ± 0.11	0.01 ± 0.13	−0.02 ± 0.11	−0.06 ± 0.17	0.264
Primary spherical (Z^0^_4_; μm)	0.16 ± 0.30	0.10 ± 0.09	0.20 ± 0.26	0.22 ± 0.54	0.739
Vetical secondary astigmatism (Z^2^_4_; μm)	0.08 ± 0.18	0.05 ± 0.16	0.03 ± 0.19	0.06 ± 0.22	0.840
Vertical quadrefoil (Z^4^_4_; μm)	−0.12 ± 0.24	−0.09 ± 0.18	−0.09 ± 0.21	−0.15 ± 0.31	0.903

*SimK* simulated keratometry, *ARK* autorefractokeratometry, *RMS* root mean square, *D* diopter; Results were presented as mean ± standard deviation.; *Statistically significant by ANOVA

## Discussion

A chalazion is a common eyelid disease, affecting individuals of all ages, caused by plugged meibomian glands and chronic lipogranulomatous inflammation [12]. Chalazia have been reported to increase corneal astigmatism and HOAs [7, 8, 13, 14]. In this study, we evaluated the effects of chalazia on the cornea according to chalazia site, location, and size using corneal topography and wavefront analysis. This study systematically revealed the mechanical effects of chalazia on corneal astigmatism. In this study, a large-sized chalazion in the whole upper eyelid induced changes in the corneal topographical and wavefront assessments. The mechanisms behind the effects of chalazia on corneal astigmatism can be suggested as follow. Firstly, with regards to the biomechanical properties of the cornea, it has been reported that its tensile strength is 3.81 ± 0.40 MPa and its stress-strain is α = 42.81 ± 11.67 and β = 2.97 ± 0.21 [15]. Compressive pressure of chalazia in excessive of these levels can induce the corneal astigmatism. In contrast, cornea under reduced strain by corneal refractive surgery (such as LASIK) may be more affected by lower pressure [9]. Secondly, lamellar orientation in human corneas has been shown to be related to mechanical properties [16, 17]. The mechanical effects increase in the meridian direction as they become closer to the center of the cornea [17]. Variations in the regional elastic performance of the human cornea have been reported; the pressure-induced meridional strains were smallest at the corneal paracenter and periphery, with the largest recorded at the limbus [18]. The circumferential strains varied less between regions with the para-centre straining to the greatest extent. In the meridional direction, Young’s modulus of elasticity was greatest at the central and para-central corneal regions, while the greatest circumferential elastic modulus was found at the limbus [17, 18]. Some authors have suggested the notion of circumferentially orientated reinforcing structures in human limbal tissue [18]. The para-central region of the human cornea was found to be stiffer in the meridional direction compared with the circumferential direction, suggesting a meridionally-orientated reinforcement of the para-central parts of the human cornea [18]. Furthermore, the human corneal stroma exhibit a preferred collagen orientation in the inferior-superior and nasal-temporal directions. However, at the limbus, the preferred orientation is tangential to the cornea [19]. Therefore, it is difficult for the pressure on the sclera to have an effect on the cornea in the meridian direction. Chalazia in the middle eyelid can more easily induce corneal astigmatism in the meridian direction because it is located superior to the cornea and close to the center of the cornea. The mass effect of a chalazion could increase with size. Chalazia generally affected Z^−2^, an aberration of off-axis rays. Furthermore, HOAs influence sensitivity to contrast to varying degrees at different orientations [20].

These findings may have implications in pediatric patients at risk of amblyopia [13]. In addition, transient chalazion-induced astigmatism can disturb the visual acuity, mislead intraocular lens calculation before cataract surgery, and result in serious error during refractive surgery. Therefore, in these cases, chalazia should be treated in the early phase. Long-term chalazia may induce the remodeling of corneal stroma through the secretion of inflammatory mediators including matrix metalloproteinases. Chalazia excision can decrease corneal astigmatism and irregularity; this is more prominent in single, firm, and central upper eyelid lesions [14]. Treatment modality includes incision and curettage, intralesional triamcinolone injection, and intralesional botulinum injection.

## Conclusions

Large-sized chalazia in the whole upper eyelid should be treated in the early phase because they induced the greatest change in corneal topography. Chalazion should be treated before corneal topography is performed preoperatively and before the diagnosis of corneal diseases.

## Additional file

Additional file 1: Dataset_1. The data for chalazion and corneal topography. Data were obtained from the review of medical charts of a total of 114 eyes from 64 patients between July 2013 and April 2015. Data included the size and location of chalazia and corneal topographic measurements in the chalazion group and control. (XLS 79 kb)

## Abbreviations

ANOVA: Analysis of variance
ARK: Autokeratorefractometer
CCT: Central corneal thickness
D: Diopter
HOA: High order aberration
K: Keratometry
RMS: Root mean square
